# IgG2 rules: N-acetyl-β-D-glucosamine-specific IgG2 and Th17/Th1 cooperation may promote the pathogenesis of acute rheumatic heart disease and be a biomarker of the autoimmune sequelae of *Streptococcus pyogenes*

**DOI:** 10.3389/fcvm.2022.919700

**Published:** 2023-02-06

**Authors:** Christine A. Kirvan, Heather Canini, Susan E. Swedo, Harry Hill, George Veasy, David Jankelow, Stanley Kosanke, Kent Ward, Yan D. Zhao, Kathy Alvarez, Andria Hedrick, Madeleine W. Cunningham

**Affiliations:** ^1^Department of Biological Sciences, California State University, Sacramento, CA, United States; ^2^Pediatrics and Developmental Neuropsychiatry Branch, National Institute of Mental Health, National Institutes of Health, Department of Health and Human Services, Bethesda, MD, United States; ^3^Departments of Pediatrics, Infectious Diseases, Cardiology, and Pathology, University of Utah College of Medicine, Salt Lake City, UT, United States; ^4^Division of Cardiology, University of Witwatersrand, Johannesburg, South Africa; ^5^Department of Comparative Medicine, University of Oklahoma Health Sciences Center, Oklahoma City, OK, United States; ^6^Department of Pediatrics, Division of Cardiology, University of Oklahoma Health Sciences Center, Oklahoma City, OK, United States; ^7^Department of Biostatistics and Epidemiology, University of Oklahoma Health Sciences Center, Oklahoma City, OK, United States; ^8^Department of Microbiology and Immunology, University of Oklahoma Health Sciences Center, Oklahoma City, OK, United States

**Keywords:** acute rheumatic fever, Th17 cells, IgG subclass, autoimmunity, group A streptococci

## Abstract

Antecedent group A streptococcal pharyngitis is a well-established cause of acute rheumatic fever (ARF) where rheumatic valvular heart disease (RHD) and Sydenham chorea (SC) are major manifestations. In ARF, crossreactive antibodies and T cells respond to streptococcal antigens, group A carbohydrate, N-acetyl-β-_D_-glucosamine (GlcNAc), and M protein, respectively, and through molecular mimicry target heart and brain tissues. In this translational human study, we further address our hypothesis regarding specific pathogenic humoral and cellular immune mechanisms leading to streptococcal sequelae in a small pilot study. The aims of the study were to (1) better understand specific mechanisms of pathogenesis in ARF, (2) identify a potential early biomarker of ARF, (3) determine immunoglobulin G (IgG) subclasses directed against GlcNAc, the immunodominant epitope of the group A carbohydrate, by reaction of ARF serum IgG with GlcNAc, M protein, and human neuronal cells (SK-N-SH), and (4) determine IgG subclasses deposited on heart tissues from RHD. In 10 pediatric patients with RHD and 6 pediatric patients with SC, the serum IgG2 subclass reacted significantly with GlcNAc, and distinguished ARF from 7 pediatric patients with uncomplicated pharyngitis. Three pediatric patients who demonstrated only polymigrating arthritis, a major manifestation of ARF and part of the Jones criteria for diagnosis, lacked the elevated IgG2 subclass GlcNAc-specific reactivity. In SC, the GlcNAc-specific IgG2 subclass in cerebrospinal fluid (CSF) selectively targeted human neuronal cells as well as GlcNAc in the ELISA. In rheumatic carditis, the IgG2 subclass preferentially and strongly deposited in valve tissues (*n* = 4) despite elevated concentrations of IgG1 and IgG3 in RHD sera as detected by ELISA to group A streptococcal M protein. Although our human study of ARF includes a very small limited sample set, our novel research findings suggest a strong IgG2 autoantibody response against GlcNAc in RHD and SC, which targeted heart valves and neuronal cells. Cardiac IgG2 deposition was identified with an associated IL-17A/IFN-γ cooperative signature in RHD tissue which displayed both IgG2 deposition and cellular infiltrates demonstrating these cytokines simultaneously. GlcNAc-specific IgG2 may be an important autoantibody in initial stages of the pathogenesis of group A streptococcal sequelae, and future studies will determine if it can serve as a biomarker for risk of RHD and SC or early diagnosis of ARF.

## Introduction

Acute rheumatic fever (ARF) is a multisystem inflammatory sequela of group A streptococcal (GAS) pharyngitis where streptococcal antigens provoke autoimmune responses in susceptible individuals and target the heart, brain, and joints (1–3) causing rheumatic heart disease (RHD), Sydenham chorea (SC), or polymigrating arthritis. A resurgence of ARF was observed in the mid-1980s in the United States and currently continues unabated in developing countries (4–11). The pathogenesis of ARF is not completely understood but is believed to be mediated by autoimmune and inflammatory mechanisms initiated by streptococcal infection where molecular mimicry generating crossreactive autoantibodies and T cell responses play a role in the clinical manifestations in a susceptible host (12–16). Diagnosis of rheumatic fever is based on revised Jones criteria that include clinical observations as well as evidence of recent streptococcal infection, identification of antistreptococcal antibodies in blood, or positive throat culture for group A streptococci (1).

Rheumatic heart disease is the most serious manifestation of ARF and a leading cause of acquired pediatric heart disease worldwide where patients develop valvulitis or myocarditis (3, 17–20). In RHD, development of valvular lesions appear to result from antibody deposition with upregulation of vascular cell adhesion molecule 1 (VCAM-1) at the surface of the valve followed by infiltration of primarily CD4+ and some CD8+ T lymphocytes that promote inflammation, fibrosis, and scarring of the valves disrupting cardiac function (21–28). T cells from RHD have been found to secrete proinflammatory cytokines, including IFN-γ and TNFα (16, 29). RHD patients develop valvulitis and heart murmur characterized by mitral and aortic regurgitation (20, 30–34). Severity of heart damage is related to the extent of valvular involvement, and injury to the heart valves may require their replacement (3).

Sydenham chorea (SC) is the principal neurologic manifestation of ARF characterized by involuntary movements and neuropsychiatric disturbances, which may develop in 10–30% of ARF cases (35–38). SC is a basal ganglia disorder which is characterized by autoantibodies that target dopaminergic neurons in the basal ganglia and activate calcium/calmodulin-dependent protein (CaM) kinase II activity as well as increased tyrosine hydroxylase production and dopamine synthesis and release in human neuronal cells (14, 39, 40). Patients with SC produce autoantibodies that recognize and signal the dopamine 2 long receptor (D2LR), which serves as a biomarker for the disorder (41–44) and accompanying rare autoimmune psychoses (45). Expression of SC derived human monoclonal antibody (mAb) V genes in transgenic mice demonstrated that the SC autoantibody targeted dopaminergic neurons in the ventral tegumental area or the substantia nigra of the basal ganglia (40).

There are many gaps in our knowledge of the stages and causes of RHD and SC. Although autoimmunity and molecular mimicry have been a hallmark of group A streptococcal sequelae for decades, there are many other important pathogenic mechanisms that may contribute to the autoimmune state either simultaneously or in stages to produce valvular heart disease severity and outcomes. These mechanisms and how they work together and produce disease are yet to be completely understood and new biomarkers identified. In addition to the mimicry between streptococci and heart, RHD may be caused or exacerbated by release of collagen from damaged tissues (3, 46), development of anti-myosin and anti-collagen antibodies (47) and collagen reactive T cells (48), as well as a fibrotic response to elevated transforming growth factor beta-1 (TGF-β1) in RHD tissues (49). To explain the left sided mitral valve association with heart valve injury, an alternate hypothesis has been proposed suggesting that initial group A streptococcal infection provokes inflammatory signaling (TNFα and IL-6), inducing epigenetic changes that prime gene expression in the endothelial and interstitial cells of cardiac valves (49). Epigenetic priming of valve tissue and exposure to hemodynamic stress attributable to transvalvular pressure gradients (TVPGs) are necessary prerequisites for the initiation and progression of valve disease. Acute valvulitis and progression to RHD preferentially occur in the valves exposed to high TVPGs (49). The valves that are normally not exposed to high hemodynamic stress only develop lesions characteristic of RHD when exposed to elevated TVPGs. TGF-β1 signaling plays a key role in initiating and sustaining the fibrosis responsible for chronic RHD. This mechanism provides an explanation for both the preferential valve involvement seen in ARF and RHD and the reactivation and progression of valve disease (49). Class II human leukocyte antigen (HLA) molecules predisposition has been proposed to affect RHD, and different HLA alleles have been reported to be associated with RHD susceptibility in different ethnic populations (50–55) while alterations at the C4 complement factor (56), TGF-β1 (49), mannose binding protein, and FcR loci may be pathogenic (13, 57). It is not clear if all of these factors work together simultaneously or if certain ones follow others. Certainly, we do know that autoimmunity and inflammation in the beginning of disease would lead to fibrosis which is an important effect of Th17 cells.

Previous studies have also found humoral mechanisms which may play a role in the pathogenesis of ARF and RHD. The Moreland laboratory has correlated an elevated IgG3-complement C4 protein inflammatory response that is distinctly elevated in ARF from a New Zealand Maori cohort compared to normal control subjects. The IgG3 response, which directs robust complement-mediated cell lysis, was elevated against the serotype specific M protein. The IgG3 response could be directed by IL-21 from an increased T_FH_ cell subset which may lead to brain and heart crossreactive autoimmune responses against the streptococcal M protein in ARF. Of interest, elevated concentrations of IL-21 were found in the mitral valves of RHD patients, indicating a potentially important role for this cytokine in group A streptococcal sequelae (58).

Our current study provides a more in depth understanding of the immunopathogenesis of ARF where GlcNAc-specific IgG2 was the dominant subclass of GAS directed pathogenic responses in RHD and SC, separating ARF from uncomplicated pharyngitis where antibodies do not cause disease. Furthermore, our study links Th17 cells/IL-17A with rheumatic carditis and demonstrates the coincidence of IL-17A and IFN-γ with IgG2 deposition, the most prominent IgG subclass on RHD derived valve tissues. Similarly, IgG2 present in CSF from rheumatic chorea recognized human neuronal cells to identify pathogenic responses in SC. In both RHD and SC, a significant and preferential sera IgG2 response recognized GlcNAc, the dominant group A carbohydrate epitope. Anti-GlcNAc IgG2, in comparison with the other subclasses, was highly elevated and distinct, and clearly separated ARF from pharyngitis. Taken together, our novel human findings suggest that IgG2 may be a pathogenic subclass in RHD and SC and that Th17/Th1 cells cooperate to play an important role in development of IgG2 in ARF. Our novel human study supports past studies of elevated immune responses against the group A carbohydrate which has been associated with a poor valvular prognosis and replacement outcomes (59).

Further, there are no disease related biomarkers for RHD. Understanding and defining new biomarkers that might be used in underdeveloped resource settings has been a worldwide goal. Understanding and defining new biomarkers that might be used in underdeveloped resource settings has been a worldwide goal. We herein define a potential serum biomarker which may identify children at risk of rheumatic fever and rheumatic heart disease and potentially explain how the response to the group A streptococcus could lead to RHD. Our hypothesis, reiterated, is that the development of an autoimmune state in RHD and SC may in part result from secretion of specific IgG subclasses directed at antigens related to carbohydrate moieties either by mimicry or direct design and such antibodies directed to tissues by specific autoreactive T helper cell subsets. The Th1 cell subset and its IFN-γ production promotes IgG2 (60–62), the IgG subclass directed against carbohydrate antigens in humans. Determination of the IgG2 subclass deposited in diseased tissues can provide insights into mechanisms of disease as well as the nature of streptococcal and host antigens that promote RHD and SC, and by their distinction and separation from uncomplicated streptococcal pharyngitis become a new potential biomarker of ARF and RHD in humans.

## Materials and methods

### Subjects, sera, CSF, RHD valve tissues and study design

Samples were obtained from patients and healthy volunteer subjects enrolled in research protocols at the University of Oklahoma Health Sciences Center, University of Utah School of Medicine, University of Witwatersrand, and the National Institutes of Health. The protocols were reviewed by institutional review boards (IRBs) at the respective institutions, and appropriate assent/consent obtained prior to collection of clinical data and samples. Specifically, the research study protocols were approved for the study of human subjects by the institutional review boards at the National Institutes of Health Combined Neuroscience Institutional Review Board, Bethesda, MD, USA; and at the University of Oklahoma Health Sciences Center Institutional Review Board for Protection of Human Subjects, Oklahoma City, OK, USA. In all studies, each parent and child gave written consent or assent, respectively, for the investigation. Parents gave written consent for their children to participate (witnessed by a member of the NIMH human subjects protection team). All children 7 years and older gave written assent to participate and those 6 and under gave verbal assent. Samples were de-identified and coded to obscure identity and diagnosis prior to laboratory storage or shipment. All serum and tissue samples from outside Oklahoma were deidentified prior to receipt by Dr. Cunningham’s laboratory and patients were not in any case identifiable by her laboratory.

Blood was collected from patients at the time of diagnosis of ARF/RHD/SC based on the revised Jones criteria for each individual patient studied. SC patients were collected and diagnosed at the NIMH and in Oklahoma University Hospital and the RHD patients were collected during the Utah ARF outbreak and diagnosed at the University of Utah College of Medicine and Hospitals and patients with ARF and RHD were diagnosed at the Oklahoma University Hospital. Table 1 describes the number of patients who are included in the study including the numbers of each type of patient, source, ages and diagnosis. Jones criteria are mentioned in Table 1. Figures also list the numbers of patients included but the scatter plots show the direct result of each patient which can be followed on the scatter plot graphs shown in the results.

**TABLE 1:** Acute rheumatic fever: Pediatric patient cohort^b^^c^.

ARF manifestation^a^^b^^c^	Number of sera	Source of sera
Carditis/RHD	10	7 sera from OUHSC clinics/hospital
		3 sera from Utah ARF outbreak
Chorea	6	1 sera from OUHSC clinics/hospital
		5 sera from the NIMH repository
Arthritis	3	1 sera from OUHSC clinics/hospital
		2 sera from Utah ARF outbreak
**Controls**		
Uncomplicated Pharyngitis^b^^d^	7	7 sera from the OUHSC clinics
Healthy Children (ASO < 150)	8	8 sera from the OUHSC clinics

^a^Diagnosis by revised jones criteria.

^b^Positive ASO titer > 200 Todd Units in pharyngitis and/or ARF pediatric groups (OUHSC serology laboratory).

^c^Age range 8–17 for pediatric ARF and pediatric control groups.

^d^Diagnosis of pharyngitis by positive throat culture and/or positive ASO titer (OUHSC serology laboratory).

RHD, polymigrating arthritis, and SC patients were assessed by the revised Jones criteria and were GAS culture positive or anti-streptolysin O (ASO) titer positive. RHD and polymigrating arthritis sera were collected in the Departments of Pediatrics, Infectious Diseases, Cardiology, and Pathology at the University of Utah College of Medicine, Salt Lake City, UT, USA. SC sera and CSF samples were collected at the National Institutes for Mental Health. Additional RHD, SC, uncomplicated pharyngitis, and healthy control sera were collected from the Department of Pediatrics, Divisions of Cardiology and Neurology at the University of Oklahoma Health Sciences Center. Sera were all pediatric cases, and ARF sera ranged from 8 to 17 years old. All serum or CSF samples were stored after collection at −135°C until testing in our assays.

Valve specimens were obtained during surgery or autopsy from 4 patients with rheumatic heart disease at the University of Witwatersrand, Johannesburg, South Africa. The patient ages ranged from 8 to 15 years old. Rheumatic valves were compared with anatomically and functionally normal mitral valves from autopsy of middle-aged individuals after myocardial infarction.

### Histology

Examination of hematoxylin-eosin (H&E) stained sections were characterized according to the cellular infiltration, fibrosis (scarring), neovascularization, and mineralization in the valves. Selected heart tissues had evidence of chronic valvulitis, defined by the presence of inflammatory cellular infiltrates of mononuclear cells and neutrophils with scarring, neovascularization, and absence of significant mineralization.

### Cell lines

The human SK-N-SH (ATCC HTB-11) neuroblastoma line was obtained from the American Type Culture Collection. Cells were routinely cultured with F12-DMEM (Gibco-BRL) media containing 10% fetal calf serum (FCS), 1% penicillin and streptomycin, and 0.1% gentamicin at 37°C, 5% CO_2_.

### Immunohistochemistry

Heart tissues were embedded with paraffin to produce slides containing 5-μm tissue sections. Tissue sections were deparaffinized in a 3:1 mixture of Hemo-D:xylene and rehydrated in graded ethanol and washed in PBS for 5 min. For studies of the cytokines in tissues, deparaffinized sections were subjected to antigen retrieval using citrate buffer (10 mM Citrate Buffer, pH 6.0 for 20 min in hot steam) (63). Sections were washed in PBS for three minutes and blocked with Protein Blocker (BioGenex) diluted 1:10 in distilled water with 5% bovine serum albumin then probed with goat anti-human IL-17A, anti-IFN-γ, or isotype control at 15 μg/ml (R&D Systems, Minneapolis, MN, USA) overnight at 4°C. Following overnight incubation, slides were washed three times for 3 min in PBS and then incubated with biotin-conjugated rabbit anti-goat IgG (1:1,000; Abcam) overnight at 4°C. After washing slides in PBS, alkaline phosphatase-conjugated streptavidin (1:1,000 in PBS; Jackson ImmunoResearch) was incubated on tissues for 30 min at room temperature. Slides were washed again in PBS three times for three minutes and antibody binding was detected with Fast Red substrate (BioGenex) (64) followed by counterstaining with Mayer’s hematoxylin (Biogenex). Non-RHD heart tissue served as a negative control. Stained tissues were read by at least 3 individuals and graded 0±, 1+, 2+, 3+, and 4+ with the range from zero or no staining or = /− or very weak staining, 1+ clear visible but light staining; 2+ or moderate staining, 3+ moderate to heavy staining, and 4 + the strongest staining visible and all read in a Olympus dual light and fluorescence microscope with computer photography work station and camera attached to the microscope.

Heart tissues from RHD were tested for heart tissue-bound antibody which was detected using biotin-conjugated rabbit anti-human IgG1, IgG2, IgG3, and IgG4 monoclonal antibodies (Sigma Chemical Co.) incubated at 4°C overnight. Tissues were washed in PBS three times for 3 min and incubated with alkaline phosphatase-conjugated streptavidin (1:1,000 in PBS; Jackson ImmunoResearch). Antibody binding was detected using Fast Red Substrate (Biogenex, San Ramon, CA, USA). Non-RHD heart tissue served as a negative control. Stained cells were read by at least three individuals and graded 0, ±, 1+, 2+, 3+, and 4+ as shown in the Figures 5–7 with the darkest staining registered as a 4+ and the weakest as ± or 1+ accordingly.

**FIGURE 1:**
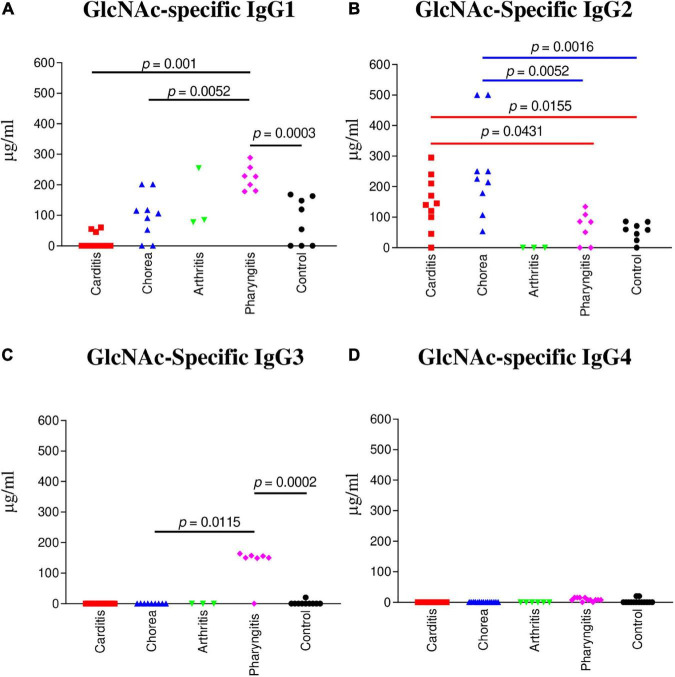
IgG subclass responses (μg/ml) to GlcNAc in ARF (RHD, SC, Arthritis) sera compared to pharyngitis and healthy control groups. **(A)** The concentration of GlcNAc-specific IgG1 was significantly elevated in uncomplicated pharyngitis sera in comparison to RHD (*p* = 0.001), SC (*p* = 0.0052), and healthy control sera (*p* = 0.0003), but not to rheumatic arthritis (*p* = 0.2667). **(B)** GlcNAc-specific IgG2 concentrations were significantly elevated in SC sera in comparison to uncomplicated pharyngitis (*p* = 0.0052) and healthy control (*p* = 0.0016) sera, but not to RHD sera (*p* = 0.0653). RHD sera had significant amounts of IgG2 to GlcNAc in comparison to uncomplicated pharyngitis (*p* = 0.0431) and healthy control sera (*p* = 0.0155). **(C)** GlcNAc-specific IgG3 in sera from uncomplicated pharyngitis was significantly elevated in comparison to SC (*p* = 0.0115) and healthy control (*p* = 0.002) sera. **(D)** GlcNAc-specific IgG4 concentrations were negligible for all groups. *P* values were calculated by the Mann-Whitney two-tailed *t* test for comparison of individual sera groups of carditis (*n* = 10) SC (*n* = 9), arthritis (*n* = 3), pharyngitis (*n* = 7), and healthy control (n = 8) (Red *p* value bars are RHD comparisons vs Blue *p* value bars are SC comparisons vs Black *p* value bars are pharyngitis comparisons). GlcNAc-specific IgG2 was the significant subclass response to GlcNAc in RHD and SC sera compared to pharyngitis and healthy controls, while GlcNAc-specific IgG1 and IgG3 were significantly elevated in pharyngitis sera. NS, not significant.

**FIGURE 2:**
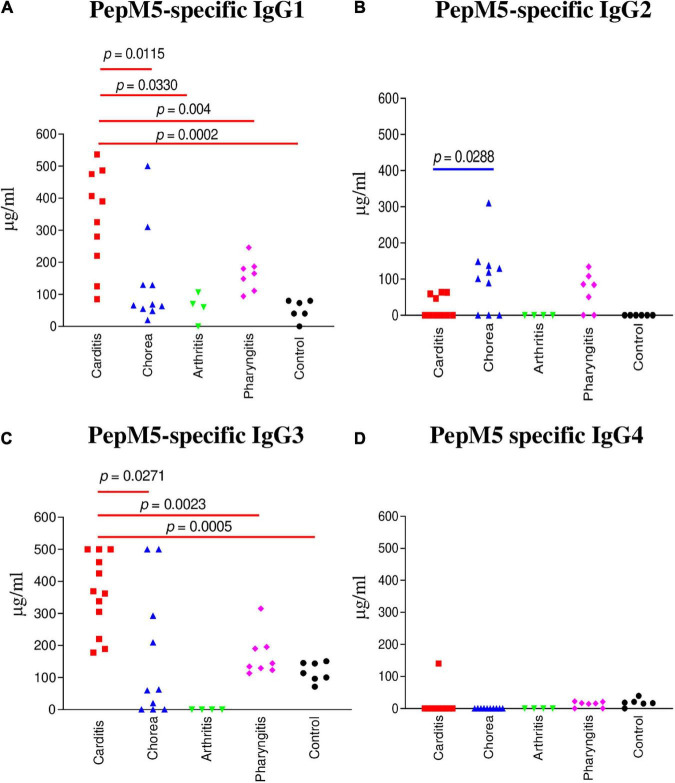
IgG subclass responses (μg/ml) to the group A streptococcal M protein type 5 (pepM5) in ARF (RHD, SC, Arthritis) sera, pharyngitis and healthy control groups. **(A)** In rheumatic carditis, IgG1 was the dominant subclass response to pepM5 in comparison to SC (*p* = 0.0115), rheumatic arthritis (*p* = 0.004), uncomplicated pharyngitis (*p* = 0.0330), and healthy control (*p* = 0.0002) sera. **(B)** PepM5-specific IgG2 was significantly elevated in SC sera in comparison to RHD samples (*p* = 0.0288). Neither SC nor RHD sera had significant amounts of anti-pepM5 IgG2 in comparison to uncomplicated pharyngitis (*p* = 0.3148 and *p* = 0.0688, respectively). **(C)** Significantly elevated pepM5-specific IgG3 was found in rheumatic carditis in comparison to SC (*p* = 0.0271), pharyngitis (*p* = 0.0023), and healthy control (*p* = 0.0005) sera. **(D)** PepM5-specific IgG4 concentrations were negligible in all samples. *P* values were calculated by the Mann–Whitney two-tailed *t* test for comparison of individual sera groups of carditis (*n* = 10) SC (*n* = 9), arthritis (*n* = 3), pharyngitis (*n* = 7), and healthy control (*n* = 8) (Red *p* value bars are RHD comparisons vs Blue *p* value bars are SC comparisons vs Black *p* value bars are pharyngitis comparisons). IgG1 and IgG3 were the significant subclass responses in sera to pepM5 in RHD while IgG2 was significant in SC. NS, not significant.

**FIGURE 3:**
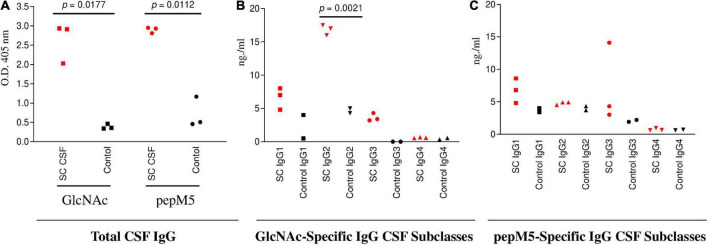
SC CSF (*n* = 3) IgG responses to GlcNAc and PepM5 GAS protein. **(A)** Total CSF IgG recognition of GlcNAc and PepM5 streptococcal protein. SC CSF demonstrated significantly elevated GlcNAc-specific IgG and pepM5-specific IgG in comparison to disease control CSF (*p* = 0.0177 and *p* = 0.0112, respectively). *P* values were calculated by the two-tailed Welch’s *t* test for comparison of SC and control CSF groups. **(B)** GlcNAc-specific IgG2 dominates SC CSF subclass responses. SC CSF demonstrates significantly higher concentrations of GlcNAc-specific IgG2 (*p* = 0.0021) in comparison to disease control CSF. The concentration of SC CSF IgG2 was significantly elevated in comparison to SC CSF IgG1 (0.01) and SC CSF IgG3 (0.0002). *P* values were calculated by the two-tailed Welch’s *t* test for comparison of SC and control CSF groups. **(C)** SC CSF IgG concentrations reactive to pepM5 show slightly elevated levels of IgG1 and IgG3 in comparison to disease control CSF, but were not significantly elevated (*p* = 0.1166 and 0.2842, respectively). *P* values were calculated by the two-tailed Welch’s *t* test for comparison of SC and control CSF groups. Red dots are SC disease CSF (*n* = 3) vs Black dots disease control CSF (*n* = 3). NS, not significant.

**FIGURE 4:**
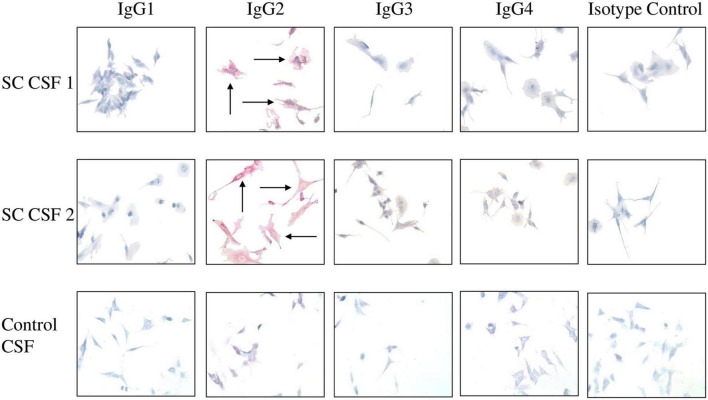
IgG2 in SC CSF targets human neuronal cells. The IgG subclasses from CSF capable of binding to surface antigen on SK-N-SH human neuronal cells were determined by Fast Red staining followed by counterstaining with hematoxylin. Red staining of cells indicates positive IgG binding. IgG2 from SC CSF predominantly targeted the extracellular surface of SK-N-SH cells (SC CSF 1 = 3 + staining and SC CSF 2 = 4 + staining) in comparison to the other subclasses (blue stained cells, 0 staining). Arrows indicate positive reactivity. Very faint IgG2 staining is shown for control CSF (0.5 + staining). In contrast, there is no IgG1, IgG3, or IgG4 binding to human neuronal cells by disease control CSF (blue stained cells, 0 staining). Magnification 400×.

**FIGURE 5:**
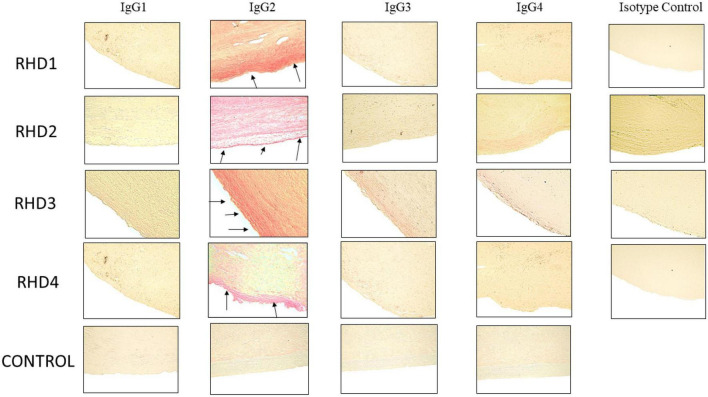
Immunohistochemistry for IgG subclasses reveals strong human IgG2 deposition in RHD heart tissues from four different patients compared to other subclasses as seen by Fast Red stain of IgG subclass deposition. Red staining of cells indicates a positive IgG binding (see arrows). RHD 1 IgG2 staining is 4+, RHD 2 IgG2 staining is 3+, RHD 3 IgG2 is 4+, RHD 4 IgG2 staining is 2+. Faint staining IgG3 (RHD 3 0.5+) and IgG4 (RHD 2 1 =, RHD 3 0.5+) staining was present. No visible IgG1 staining was observed for any of the four RHD samples. IgG subclass deposition is absent from Isotype control and non-RHD heart tissue (CONTROL). Magnification 400×.

**FIGURE 6:**
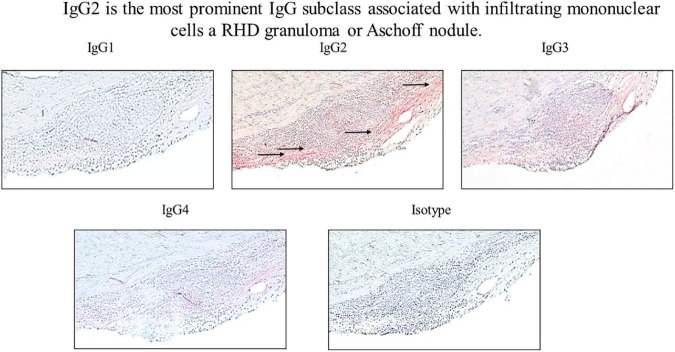
IgG2 is associated with infiltrating mononuclear cells in RHD granuloma. Elevated levels of autoreactive IgG2 were found in association with a mononuclear cell infiltrates in an RHD cardiac valvular tissue granuloma (Aschoff nodule). IgG2 deposition was qualitatively higher in comparison to other IgG subclasses at the valve surface where the granuloma was present (IgG1 0 staining, IgG2 4 + staining, IgG3 2 + staining, IgG4 1 + staining, isotype control 0 staining). Magnification 200×.

**FIGURE 7:**
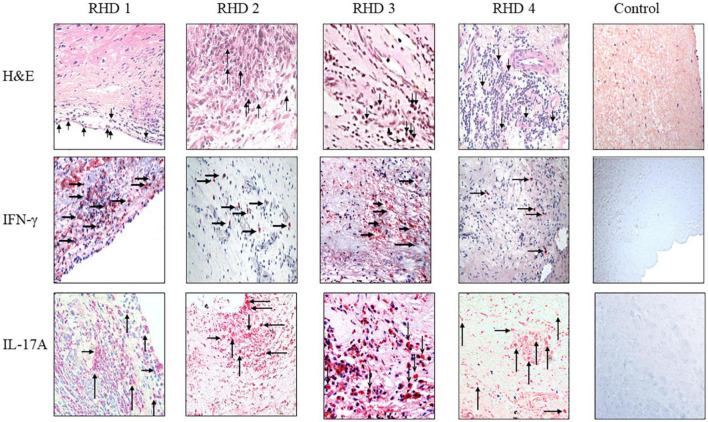
IgG2 is associated with IFN-γ and IL-17A in RHD hearts. **(Top row)** hematoxylin and eosin (H&E) staining of four RHD heart tissues showed infiltration of mononuclear cells (arrows in top row) into the heart at the valve surface endothelium (RHD 1) and within the myocardial tissues (RHD 1-4). Focal lesions (large arrow) were observed within the myocardium of RHD4. Non-RHD valvular heart tissue (control) lacked mononuclear cell infiltration and focal lesions. **(Middle row)** IFN-γ staining of RHD cardiac tissues demonstrates widespread IFN-γ staining of heart tissues was found for RHD1, and RHD3 tissues. Concentrated IFN-γ staining can be seen around invading mononuclear cells (RHD 1-RHD 4). IFN-γ is absent in the control heart tissue. Magnification 400×. **(Bottom row)** IL-17A staining of RHD cardiac tissues. Four RHD tissues shows IL-17A throughout the heart tissues as well as concentrated around mononuclear cells (arrows) (RHD 1-RHD 4). The control, non-RHD heart tissue had no IL-17A present. Magnification 400×.

To determine if the CSF bound to human neuronal cells, SK-N-SH cells, a human neuronal cell line, was plated in four-chamber tissue culture slides at 1 × 10^4^ cells/chamber overnight in DMEM-F12 medium under standard cell culture conditions. The four-chamber slides were washed with ice cold PBS and blocked with 5% BSA in PBS then incubated with a 1:100 dilution of CSF in DMEM-F12 medium overnight at 4°C. The slides were then washed in PBS and cell-bound antibody was detected using biotin-conjugated rabbit anti-human IgG1, IgG2, IgG3, and IgG4 monoclonal antibodies (Sigma Chemical Co.) for 30 min at 37°C followed by alkaline phosphatase-conjugated strepavidin (1:1,000; Jackson ImmunoResearch). Antibody binding was detected using Fast Red Substrate (Biogenex, San Ramon, CA, USA) and cells counterstained with Mayer’s hematoxylin (Biogenex). Non-SC disease CSF served as a negative control. Stained tissues were read by at least three individuals and graded 0, ±, 1+, 2+, 3+, and 4+ as shown in Figure 4 with the darkest staining registered as a 4+ and the weakest as −/− or 1+ accordingly and as described in the Methods Immunochemistry section.

### Indirect enzyme-linked immunosorbant assay (ELISA)

Pepsin fragment of streptococcal M5 protein (pepM5) (65, 66) and GlcNAc-BSA antigens were made as previously described (67–69). Purified human IgG1, IgG2, IgG3, and IgG4 antibodies were purchased from Sigma Chemical Co. (St. Louis, MO, USA).

Ninety-six well polyvinyl, Immunolon 4 microtiter plates (Dynatech Laboratories, Chantilly, VA, USA) were coated with 10 μg/ml of pepM5 or GlcNAc-BSA or 50 μg/ml of mouse anti-human IgG1, IgG2, IgG3, and IgG4 mAbs for standard curve overnight at 4°C. Plates were incubated overnight at 4°C with 1:10 dilution CSF, 1:1,000 dilution of sera, or 500 to 0 μg/ml concentrations of human IgG1κ, IgG2κ, IgG3κ, and IgG4κ purified monoclonal antibodies (Sigma Chemical Co.) to obtain a standard curve. Antibody binding was detected using biotin-conjugated rabbit anti-human IgG1, IgG2, IgG3, and IgG4 monoclonal antibodies (Sigma Chemical Co.) followed by alkaline phosphatase-conjugated strepavidin (Jackson ImmunoResearch, West Grove, PA, USA). Plates were developed with two mg/ml p-nitrophenyl phosphate 104 substrate (Sigma Chemical Co.) at 100μl/well and optical density quantified at 405 nm in an Opsys MR microplate reader (Dynex Technologies, Chantilly, VA, USA). IgG subclass concentrations of the sample reactivity in the ELISA was determined from the subclass standard curves and reported as μg/ml after BSA reactivity was deducted. Healthy control sera and non-SC disease CSF served as negative controls for ELISA. Reactivity of IgG subclass antibodies with GlcNAc and bovine serum albumin (BSA) were calculated separately and any reactivity with BSA alone subtracted from the overall optical density of each sample.

### Statistical analyses

IgG subclass responses to GlcNAc and PepM5 in ARF (RHD, SC, Arthritis) sera (Figures 1, 2) were compared to pharyngitis or healthy controls using the Mann–Whitney test. The Mann–Whitney test was chosen due to the small sample size and skewed distribution of the responses. Adjusted analysis was not deemed necessary due to small sample size and relative balance in demographic variables among individual groups. In Figure 3, Welch’s t test was used for CSF ELISAs as the Mann–Whitney was not sufficient to determine the *p* values for comparisons between the smaller numbers of normal CSF and SC CSF sample values. All statistical analyses were performed using the PRISM software and a *p*-value of <0.05 was considered significant.

## Results

### Serum IgG subclass response in acute rheumatic fever: IgG2 dominates the response against the group A carbohydrate epitope, N-acetyl-beta-D-glucosamine (GlcNAc) in RHD and SC

To determine which IgG subclass dominated humoral responses against the group A streptococcal carbohydrate in ARF, sera from RHD, SC, polymigrating arthritis, uncomplicated pharyngitis, and healthy controls were examined for GlcNAc immunoreactivity. In Figure 1B, both RHD and SC sera demonstrated significantly higher GlcNAc-specific IgG2 antibody responses in comparison to uncomplicated pharyngitis (RHD *p* = 0.0431, SC *p* = 0.0052) and to healthy control sera (RHD *p* = 0.0155, SC *p* = 0.0016) (Figure 1B). In contrast, pharyngitis sera demonstrated significantly elevated GlcNAc-specific IgG1 responses in comparison to healthy controls (*p* = 0.0003), RHD (*p* = 0.001), and SC (*p* = 0.0052) sera (Figure 1A). Of note, GlcNAc-reactive IgG3 (Figure 1C) was significantly elevated in pharyngitis sera compared to healthy control sera (*p* = 0.0002) and SC sera (*p* = 0.0115). Detectable concentrations of GlcNAc-specific IgG3 were not present in RHD or polymigrating arthritis sera. The finding was of interest as IgG1 and IgG3 typically are induced by protein antigens and are characterized by an increased ability to direct complement activation and opsonization in comparison to IgG2 (70). IgG4 was not elevated to GlcNAc in any of the sera tested. Thus, IgG2 appears to characterize RHD and SC sera as a disease-specific response against the carbohydrate epitope GlcNAc in ARF.

IgG subclass specificity was also determined for the group A streptococcal M protein fragment, pepM5, the extracellular, N-terminal half of the M5 protein cleaved by pepsin at suboptimal pH (65, 66). IgG subclass responses to pepM5 protein were distinct from those against the group A carbohydrate epitope GlcNAc (Figure 2). In rheumatic carditis, pepM5 protein-reactive IgG1 (Figure 2A) responses were significantly elevated above SC (*p* = 0.0115), arthritis (*p* = 0.0330), uncomplicated pharyngitis (*p* = 0.004), and control sera (*p* = 0.0002). RHD IgG3 (Figure 2C) concentrations were also significantly higher (*p* = 0.0271) to SC, pharyngitis (*p* = 0.0023) and healthy controls (*p* = 0.0005). In contrast, in SC, IgG2 responses against pepM5 were significantly higher in comparison to RHD (*p* = 0.0288) (Figure 2B). IgG4 was not elevated to PepM5 protein in any sera tested. Evidence provided in Figures 1, 2 show that IgG subclass responses to streptococcal antigens distinguishes ARF from uncomplicated pharyngitis and highlights GlcNAc-reactive IgG2 as a feature of both RHD and SC, which are distinct from the anti-GlcNAc IgG1 and IgG3 responses of uncomplicated pharyngitis. Furthermore, the IgG responses to GlcNAc are distinct from those to the M protein, which is shown in Figure 2 to be significantly elevated in carditis compared to pharyngitis. Interestingly, the IgG subclass response to M protein in SC was definitively IgG2 compared to carditis.

### IgG2 from SC CSF preferentially binds to GlcNAc and targets human neuronal cells

To evaluate CSF reactivity to streptococcal antigens, total IgG specificity to GlcNAc and the pepM5 protein was tested by ELISA. Significantly elevated levels of CSF total IgG to GlcNAc (*p* = 0.0177) and pepM5 (*p* = 0.0112) were found in SC CSF (Figure 3A) in comparison to CSF samples from other neurological disorders (Black dots; Figure 3). Evaluation of IgG subclass distribution to the streptococcal antigens showed significantly increased concentrations of GlcNAc-specific IgG2 in SC CSF compared to disease control CSF (*p* = 0.0021) (Figure 3B). The concentration of anti-GlcNAc IgG2 is SC CSF was also significantly higher than the concentrations of IgG1 or IgG3 from SC CSF. In Figure 3C, SC CSF IgG concentrations reactive to pepM5 show slightly elevated concentrations of IgG1 and IgG3 in comparison to disease control CSF, but were none were significantly elevated (*p* = 0.11 and 0.28, respectively).

To demonstrate which IgG subclasses potentially target neurons, SC and disease control CSF samples were incubated with SK-N-SH human neuronal cells and probed with antibody specific to the four IgG subclasses. Autoreactive IgG2 was found to qualitatively bind to the neuronal cell surface to a much greater degree than IgG1, IgG3, and IgG4 in both SC CSF samples (Figure 4). IgG2 staining was largely absent from the disease control CSF (Figure 4 control, bottom panel). Interestingly, IgG2 was the dominant subclass bound to the neuronal cell surface even though the CSF ELISA results (Figure 4) showed detectable concentrations of IgG1 and IgG3. IgG complement-mediated cytotoxicity against SK-N-SH cells were all below 10% in chromium release assays (data not shown). The data suggest that in SC-derived CSF IgG2 dominated the antibody response to streptococcal group A carbohydrate epitope GlcNAc, and autoreactive IgG2 qualitatively bound (red stain SC CSF 1 = 3+, SC CSF = 4+) to human neuronal cells substantially more than other subclasses in SC CSF.

### Autoreactive IgG2 is the dominant subclass deposited on RHD cardiac valve tissues

To determine subclass specificities of autoreactive IgG involved in RHD, cardiac tissues from valve replacement surgery or autopsy were tested for bound IgG1, IgG2, IgG3, and IgG4. Pronounced IgG2 staining of RHD cardiac valve tissues was observed in all four RHD tissues and IgG2 was distinctly overrepresented in comparison to other IgG subclasses (Figure 5). A small amount of IgG3 was evident on valvular tissues compared to the IgG2, with very little IgG1 or IgG4 staining observed. No IgG subclass deposition was observed on control heart tissue. IgG2 deposits in RHD valvular tissues clearly stood out and emphasized that anti-GlcNAc IgG2, which was significantly elevated in RHD sera, may directly deposit and contribute to carditis in RHD. Although there were significantly elevated serum concentrations of IgG1 and IgG3 to pepM5 from RHD sera, only weak IgG3 staining was visible on the heart tissues. Collectively, the data suggest that autoreactive IgG2 preferentially binds to valvular heart tissues in RHD and may contribute to cellular infiltration and inflammatory valvular changes seen in rheumatic carditis. The previous findings that elevated responses to the group A carbohydrate antigen correlated with severity and poor outcomes in valve disease supports our findings (59).

### Th17/Th1 cells infiltrate the heart valve and coincide with IgG2 deposition in rheumatic carditis

To determine if autoreactive IgG2 deposition occurred at the site of cellular pathology, RHD tissue containing a prominent granuloma (Ashoff nodule) was tested for IgG subclass staining. Strong IgG2 deposition was observed at the site of the granuloma with lesser amounts of IgG3 and IgG4 seen (Figure 6). The four RHD heart tissues with elevated IgG2 deposition all had pronounced mononuclear cell infiltrates as seen in hematoxylin and eosin-stained tissues (Figure 7 top row), which was absent in non-RHD control heart tissue (Figure 7 top row).

IFN-γ is associated with isotype switch events in B cells that leads to IgG2 secretion, and has been reported in RHD heart lesions and T cells or clones isolated from RHD hearts as well as from cardiac tissues from the Lewis rat experimental autoimmune valvulitis (EAV) model (29, 71–73). All four RHD tissues were found to have elevated levels of IFN-γ in comparison to control non-RHD heart tissue (Figure 7 middle row). Th17 cells have been reported to contribute to the pathology of myocarditis and a previous report has indicated the presence of Th17 cells in RHD hearts (64, 74). To determine if Th17 cells were present in rheumatic carditis valve tissues, IL-17A was evaluated by immunohistochemistry. IL-17A was seen throughout all four RHD myocardial and valvular tissues suggesting autoreactive Th17 cells invade into valve and myocardial tissues (Figure 7 bottom row). No IL-17A was observed in control non-RHD tissue (Figure 7 bottom row). In this study, we demonstrate that autoreactive IgG2, IFN-γ, and IL-17A was found simultaneously in the RHD tissues evaluated. Collectively, the data suggest that autoreactive IgG2 along with cooperative Th1/Th17 responses contribute to the development and progression of RHD.

## Discussion

Previously, our studies have focused on understanding the immunologic crossreactivity and molecular mimicry between GAS and heart and brain tissue antigens (14, 15, 21). In the current study, new evidence shows deposition of IgG2 on RHD cardiac tissues and suggests that antibody targeted the valves concomitant with infiltration by autoreactive Th17/Th1 T cell subsets. Additionally, in SC, GlcNAc-specific IgG2 concentrations from SC-derived CSF were significantly higher than IgG1, IgG3, and IgG4, and IgG2 was the predominant subclass that recognized cell surface antigen on human neuronal cells. Our evidence suggests IgG2 antibodies against the group A carbohydrate epitope GlcNAc as a disease specific antibody or biomarker in ARF which targets valve in RHD and human neuronal cells in SC. The elevated IgG2 response to GlcNAc was unique to RHD and SC sera in comparison to polymigrating arthritis and uncomplicated pharyngitis. In RHD, persistently high levels of anti-GlcNAc directed against heart tissues may lead to predisposition of the valves to cellular in filtration via opsonization after which the valves have the potential to upregulate fibrosis due to the IL-17A response as well as TGF-β1 genetic variations in RHD (49). IgG2 was so strongly deposited compared to other subclasses on valvular surface, that in some cases, it could be observed on some tissues with the naked eye after immunostaining. In SC CSF, only the IgG2 subclass substantially bound to the human neuronal cells. Of interest was the significantly elevated concentrations of GlcNAc-specific IgG1 and IgG3 in uncomplicated pharyngitis sera in comparison to RHD and SC sera. Both IgG1 and IgG3 strongly direct both complement activation and opsonization-phagocytosis. It may be that the secretion of IgG1 and IgG3 leads to rapid removal of GAS during uncomplicated pharyngitis, thus decreasing the potential for the development of sequelae.

While we propose mechanisms in a hypothesis where GlcNAc-specific IgG2 could play a dominant role in RHD to direct the T cell subsets to the valve, IgG3 cannot be discounted and must be considered for its potential role in RHD where IgG3 would strongly direct complement-mediated cytotoxicity, an effector function largely absent from IgG2 (75). Previously, we demonstrated endothelial cell cytotoxicity by RHD-derived human mAbs which were strongly crossreactive with (1) GlcNAc and laminin as well as (2) a highly crossreactive laminin peptide and (3) the valve surface endothelium (15). We can speculate that early production of cardiac-specific IgG3 would initiate damage at the valve leading to pronounced inflammation with edema of the valve leaflets that in turn may be replaced by a sustained IgG2 deposition and concomitant cellular infiltration with IFN-γ and IL-17A directing inflammation that characterizes RHD. The early edematous damage to valve leaflets is demonstrated in our RHD Lewis rat autoimmune model of valvulitis (73). Additional studies of the valvulitis model will be instrumental in identifying the subclasses and T cell subsets associated with initiation and persistence of heart damage.

GlcNAc-specific IgG2 was significantly lower in pharyngitis compared to RHD and SC sera, making it a potential biomarker of disease. Elevated as well as persistent IgG2 concentrations from repeated streptococcal infections may begin the inflammatory cascade at the valve endothelial surface for further injury by inflammatory T cell subsets Th1 and Th17 and the potential for enhanced fibrosis in the presence of the TGF-β1 gene variants found in RHD (49). The surface of the valve some years ago was shown to express carbohydrate epitopes (76) similar to GlcNAc, and it is well established that laminin is a glycosylated protein.

The contributions of autoreactive IgG2 to RHD is not completely clear. IgG2 is efficient in directing opsonization and can recruit macrophages, DCs, and neutrophils through the FcγRIIa receptor (CD32a), however, it is a poor activator of the complement cascade (75). In this study, IgG2 was the dominant subclass bound to RHD tissues. Previously we have shown an upregulation of VCAM-1 on cardiac valvular tissues (24). The same heart tissues shown herein were used for the VCAM-1 and IgG2 studies, thus, it is clear that VCAM-1 upregulation occurs concomitantly with IgG2 deposition with infiltration of immune cells into the valve leading to fibrosis and valve deformity. Failure to follow preventive antibiotic treatment regimens to prevent continued repeated streptococcal infection in ARF may allow for disease to intensify in the valve when there is a subsequent GAS infection (24). Thus, IgG2 may specifically contribute to each episode of cardiac damage by directing macrophages, DCs, and neutrophils into heart tissues to promote inflammation and further valve damage with epitope spreading suggested (77). Infiltration of CD68 + macrophage and CD80 + DCs correlated with increased tenascin-C concentrations, a marker for extracellular matrix remodeling and fibrosis in RHD hearts (78, 79).

In IgG2+ RHD tissues, cellular infiltration by IFN+ and IL-17A+ cells suggested a potential pathogenesis by Th17/Th1, which may involve Th17 plasticity (80), which raises questions about the relationship between Th17 and Th1 (81, 82). In autoimmune disease, Th1 cells may evolve to become autonomous and responsive directly to antigen presentation of peptides from local tissue (83) whereas in acute RHD/ARF the Th1 cells must be responding to the streptococcal and local crossreactive antigens due to molecular mimicry and have not reached the autonomous state where they would be responsive primarily to self-antigens potentially later in disease. Unlike other autoimmune diseases which wax and wane, penicillin treatment prevents progression of severe disease in ARF in the first several years after onset by preventing repeated streptococcal infections and further autoimmunity in the susceptible host (84). However, chronic valve disease may not be as responsive to preventative antibiotic therapy (85) and prevention due to hemodynamic stress attributable to transvalvular pressure gradients (49) on already damaged valves which may be targeted by immune responses against released collagen (48).

Th17 cells have been closely associated with autoimmunity (86–90). GAS have been shown to induce a robust Th17 response in mice and humans which protects against upper respiratory tract and mucosal infections and can lead to autoimmunity (64, 74, 90–103). In our current study herein, RHD hearts were infiltrated with both IL-17A and IFN-γ producing mononuclear cells concomitant with IgG2 deposition. In an earlier study, mitral valve tissues from RHD demonstrated increased expression of IL-6, IL-17, IL-21, and IL-23, and in human autoimmune myocarditis, Th17 cells secreted both IL-17A and IFN-γ in the presence of IL-23 (64, 74).

The evidence suggests a potential role of GlcNAc specific antibodies and their association with poor prognosis of valvular heart disease and outcomes in acute RHD (59). We hypothesize that the IgG2 targeting of the valve is dependent on the carbohydrate epitope and involves mimicry between the streptococcal GlcNAc and the valve surface glycoproteins in the basement membrane and valvular endocardium. Laminin contains the alpha helical structure which crossreacts with human RHD-derived human mAbs reactive with the group A streptococcal carbohydrate epitope GlcNAc and alpha helical coiled coil streptococcal M protein (15, 76, 104–109). The IgG2 deposition on valve and heart tissues and elevated anti-GlcNAc antibodies in RHD sera combined with a lower GlcNAc-specific IgG2 response in pharyngitis suggests disease specificity and ultimately IgG2 may be responsible for directing infiltration of Th1 and Th17A cell subsets as well as potential cytotoxic IgG3 responses to the valve. We propose there may be abnormally high concentrations of GlcNAc-specific IgG2 production against the GAS in RHD and SC phenotypes similar to the very high levels of anti-GlcNAc responses reported in Balb/c mice immunized with group A carbohydrate antigen. Significantly higher immune responses were seen to GlcNAc in BALB/c mice than in lower responders C57BL/6, CBA, and DBA/2 mice (110, 111).

Interferon gamma is known to signal B cell isotype switch events to the IgG2 subclass (61). In the current study, IFN-γ was found in all RHD hearts tested, and was previously reported in the pathogenesis of RHD (29, 71, 112, 113). Human T cell clones were also found to secrete a Th1 pattern of cytokines when stimulated with group A streptococcal antigens such as the M protein (16, 25, 29, 114, 115). Further, the Lewis rat model of RHD immunized with recombinant M5 protein developed myocarditis and valvulitis characterized by secretion of high levels of IL-17A and IFN-γ suggesting that the two cytokines are together important in the pathology of RHD (116).

Transition of Th17 to non-classic Th1 cells (CD161+/CCR6+) is believed to be related to the development of the autonomous responsive state of Th1 cells, which after transitioning from Th17 to Th1, respond to self-antigens presented in the attack organ leading to tissue damage (83, 117–121). This could direct responses to become more valve specific as disease progresses (48). In our previous studies, CD4+ T lymphocytes were more abundant in RHD hearts and were in the Aschoff lesions in valves, however CD8 + lymphocytes were also present and are known to also produce IFN-γ. The specific role for CD8 + T cells in RHD has been unknown in valvular heart disease (24). In recent studies, cytotoxic CD8 + T cells isolated from chronic adult human RHD valve lesions recognized collagen and identical sequence from the collagen-like streptococcal protein. The CD8+ T cell expression of granzyme and perforin as well as estrogen receptor alpha and HLA I were promoted by prothymosin alpha, a new potential blood and tissue biomarker described in RHD (48). The study emphasized valve disease primarily in women and the importance of mimicry with streptococcal or other bacterial collagen sequences (48). Previous studies have been primarily limited to CD4 + T cells in RHD with the acknowledgment that CD8+ T cells were present but not in the same numbers as the CD4+ T cells (24). Gender bias in chronic RHD in women is further supported by the identification of a dominant shared antibody idiotype found in RHD and shared with systemic lupus erythematosus (SLE) and Sjogrens syndrome (122), both systemic autoimmune diseases primarily in women where the heart can also be affected.

Molecular mimicry is important in the initiation of disease with the onset of autoimmunity. Following the development of autoimmunity, a more chronic disease course in the valves may develop at different rates in different individuals due to the number of repeated infections, hemodynamic sheer stress to the valves, as well as genetic predispositions to susceptibility to disease. These disease susceptibility factors may be genetic and include increased levels of TGF-β1 production (49), HLA (50–55), and exposure of other heart or tissue proteins (77) involved in epitope spreading with tissue antigen recognition by Th1 cells which may dominate in more chronic disease in the heart. If IgG2 continues to follow the course of deposition on the valves concomitant with exposure of collagen (3, 46), elevated TGF-β1 (49), and fibrosis with scarring and neovascularization from the damaged valves, further damage in later stages of chronic disease may be in part by CD8 + T cells and their recognition of collagen (37). Clearly, inflammation leads to fibrosis.

SC, the brain manifestation of ARF, is characterized as an antibody driven basal ganglia encephalitis (40, 41, 123–130), and the main pathologic finding is perivascular cuffing of vessels and deposition of antibody in the basal ganglia (129, 131). The role of Th17 cells in SC as well as other group A streptococcal autoimmune sequelae in humans remains obscure. However, in the murine model of GAS infection, Th17 cells found in the nasal-associated lymphoid tissue (NALT) migrated through the olfactory bulb into the CNS following intranasal GAS challenge (99). While no GAS cells were found in the CNS, the evidence strongly suggested that IL-17A disrupts the blood brain barrier (BBB) allowing crossreactive antibodies access to the brain. In murine GAS-induced autoimmune encephalitis, CNS-infiltrating Th17 cells secreting IL-17A and IFN-γ were required for the disruption of the BBB that allowed for the transit of antibody and microglial activation leading to neuropathological changes in experimental animals (132). Similar mechanisms may disrupt the BBB in SC allowing for passage of autoreactive antibody and T cells.

In SC and related disorders, brain imaging studies showed enlargement of the basal ganglia suggesting an inflammatory process (37, 38, 124, 125, 133, 134). Anti-neuronal autoantibodies found in SC and the related pediatric autoimmune neuropsychiatric disorders associated with streptococcal infections (PANDAS) which target dopamine receptors may cause the characteristics of these disorders and serve as biomarkers of disease (3, 12, 40, 41, 135). Collectively, the pathology of SC is indicative of non-cytotoxic autoantibody-mediated basal ganglia encephalitis and supports an IgG2 etiology where the crossreactive antibodies against the group A carbohydrate antigen GlcNAc lead to aberrant signaling of the neurons rather than complement-mediated cytotoxic destruction in the basal ganglia (14).

Limitations of our study include the small number of tissue and sera samples for this pilot study, and we did not perform any studies of crossreactivity except with the bacterial carbohydrate antigens. Further, many of our previous studies of ARF-derived human mAbs have demonstrated crossreactivity between GlcNAc and autoantigens including cardiac myosin and laminin in heart as well as lysoganglioside, tubulin, and dopamine receptors in human brain tissue or neurons (14, 15, 39, 40, 44, 73, 136). Although we do not have human mAbs of the IgG2 subclass to investigate, the human ARF derived mAbs which react with GlcNAc are the strongest evidence linking the anti-streptococcal GlcNAc antibodies (serum and CSF IgG and human mAbs) with crossreactivity to heart and brain.

In summary, our study suggests a new potential biomarker for ARF, RHD, and SC which can raise understanding of ARF pathophysiology. GlcNAc specific IgG2 was defined as the dominant IgG GlcNAc specific subclass in both RHD and SC in both tissues and blood, which adds to our basic understanding of group A streptococcal sequelae and pathogenesis. Further, GlcNAc-specific IgG2 distinguishes and separates the humoral IgG subclass responses of uncomplicated pharyngitis from ARF (RHD and SC). Th1/Th17 cells found in RHD and SC may cooperate to cause organ specific autoimmune T cell disease responses in the heart and brain and Th1 IFN-γ responses would promote switching the IgG subclass to IgG2. Disease specific IgG2 immune responses to GlcNAc in RHD and SC is supported by many of our previous studies demonstrating heart and brain crossreactive IgG autoantibodies and human mAbs from RHD and SC that recognize the GlcNAc epitope of the streptococcal group A carbohydrate. The novel discovery that increased levels of GlcNAc-specific IgG2 defines ARF (RHD and SC) raises our level of understanding of ARF pathophysiology as well as management of streptococcal vaccines and their safety, the future development of novel diagnostics and therapeutic strategies to identify risk and early disease, and to identify, manage, and protect against severe and chronic heart valve damage or neuropsychiatric disability in ARF. Future studies of patients from worldwide cohorts are needed to confirm our “GlcNAc-specific IgG2” hypothesis presented here and supported by our evidence.

## Data availability statement

The raw data supporting the conclusions of this article will be made available by the authors, without undue reservation.

## Ethics statement

University of Oklahoma Health Sciences Center, University of Utah School of Medicine Internal Review Boards and the NIMH Internal Review Board reviewed and approved all protocols for the study of human subjects analyzed in this manuscript. Written informed consent to participate in this study was provided by the participants’ legal guardian/next of kin.

## Author contributions

MC and CK wrote the manuscript and designed the experiments, graphs and diagrams. AH, HC, CK, and KA performed experiments for the manuscript. MC, KA, and CK were responsible for design and production of the figures for the manuscript. HH and GV provided patient samples from ARF outbreak and provided discussions and clinical information for the study and HH assisted with the manuscript. DJ provided tissues samples from RHD patients from South Africa and assisted with the manuscript. SK performed the pathology studies and assisted with the manuscript. SS provided well characterized clinical samples of serum and cerebrospinal fluid from Sydenham chorea patients and assisted with the manuscript. YZ provided expert statistical analysis and writing of the manuscript. All authors contributed to the article and approved the submitted version.
