# Corrigendum: IgG2 rules: N-acetyl-β-D-glucosamine-specific IgG2 and Th17/Th1 cooperation may promote the pathogenesis of acute rheumatic heart disease and be a biomarker of the autoimmune sequelae of *Streptococcus pyogenes*

**DOI:** 10.3389/fcvm.2023.1267920

**Published:** 2023-08-31

**Authors:** Christine A. Kirvan, Heather Canini, Susan E. Swedo, Harry Hill, George Veasy, David Jankelow, Stanley Kosanke, Kent Ward, Yan D. Zhao, Kathy Alvarez, Andria Hedrick, Madeleine W. Cunningham

**Affiliations:** ^1^ Department of Biological Sciences, California State University, Sacramento, CA, United States; ^2^ Department of Health and Human Services, Pediatrics and Developmental Neuropsychiatry Branch, National Institute of Mental Health, National Institutes of Health, Bethesda, MD, United States; ^3^ Departments of Pediatrics, Infectious Diseases, Cardiology, and Pathology, University of Utah College of Medicine, Salt Lake City, UT, United States; ^4^ Division of Cardiology, University of Witwatersrand, Johannesburg, South Africa; ^5^ Department of Comparative Medicine, University of Oklahoma Health Sciences Center, Oklahoma City, OK, United States; ^6^ Department of Pediatrics, Division of Cardiology, University of Oklahoma Health Sciences Center, Oklahoma City, OK, United States; ^7^ Department of Biostatistics and Epidemiology, University of Oklahoma Health Sciences Center, Oklahoma City, OK, United States; ^8^ Department of Microbiology and Immunology, University of Oklahoma Health Sciences Center, Oklahoma City, OK, United States

**Keywords:** acute rheumatic fever, Th17 cells, IgG subclass, autoimmunity, group A streptococci

A Corrigendum on IgG2 rules: N-acetyl-β-D-glucosamine-specific IgG2 and Th17/Th1 cooperation may promote the pathogenesis of acute rheumatic heart disease and be a biomarker of the autoimmune sequelae of *Streptococcus pyogenes* by Kirvan CA, Canini H, Swedo SE, Hill H, Veasy G, Jankelow D, Kosanke S, Ward K, Zhao YD, Alvarez K, Hedrick A and Cunningham MW (2023). Front. Cardiovasc. Med. 10: 919700. doi: 10.3389/fcvm.2022.919700

In the published article, there was an error in Figure 5 as published. One of the original figures was accidentally replicated and the correct figure left out. The corrected Figure 5 and its caption appear below.

**Figure 5:**
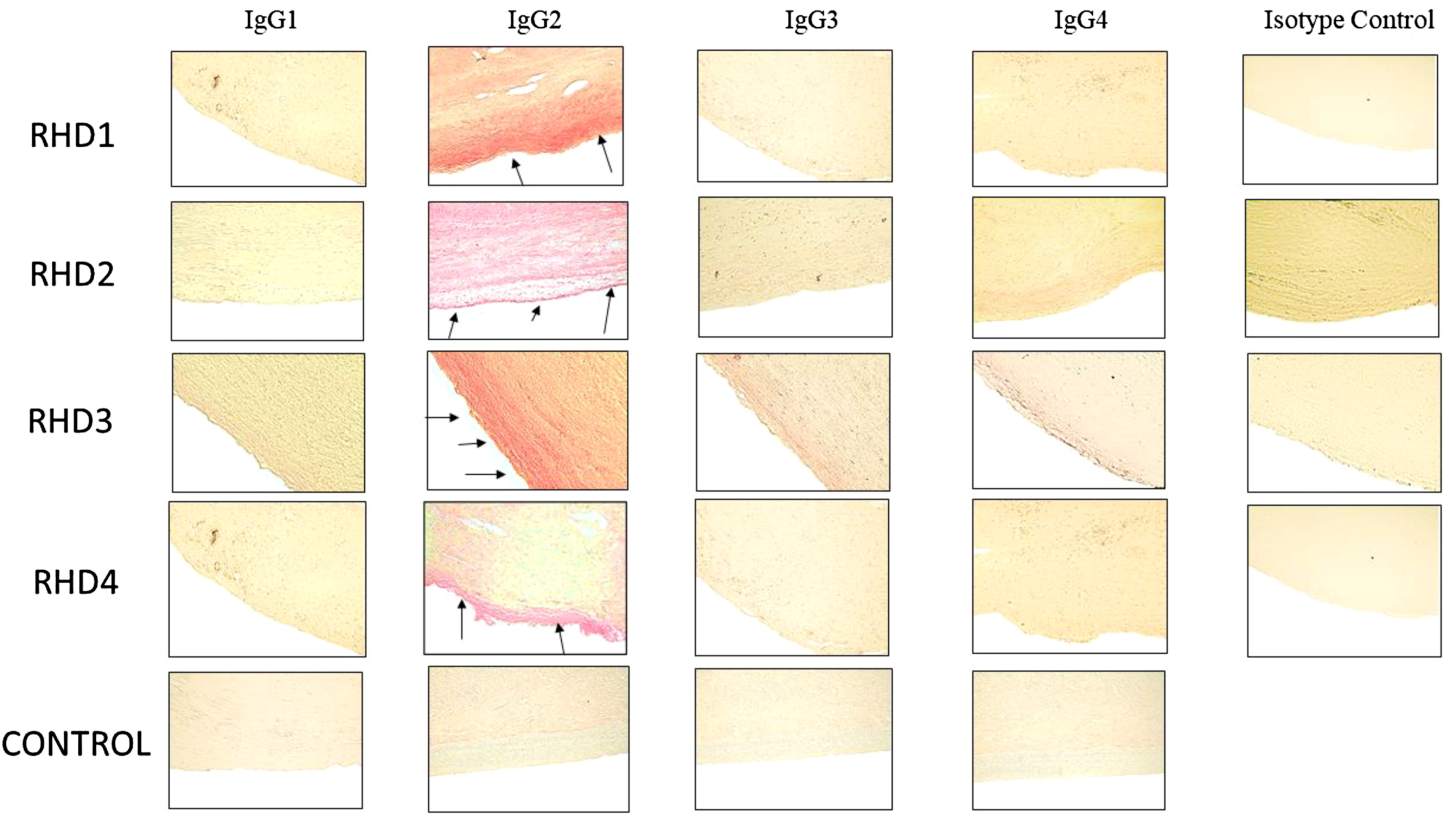
Immunohistochemistry for IgG subclasses reveals strong human IgG2 deposition in RHD heart tissues from four different patients compared to other subclasses as seen by Fast Red stain of IgG subclass deposition. Red staining of cells indicates a positive IgG binding (see arrows). RHD 1 IgG2 staining is 4+, RHD 2 IgG2 staining is 3+, RHD 3 IgG2 is 4+, RHD 4 IgG2 staining is 2+. Faint staining IgG3 (RHD 3 0.5+) and IgG4 (RHD 2 1 =, RHD 3 0.5+) staining was present. No visible IgG1 staining was observed for any of the four RHD samples. IgG subclass deposition is absent from Isotype control and non-RHD heart tissue(CONTROL). Magnification 400X.

The authors apologize for this error and state that this does not change the scientific conclusions of the article in any way. The original article has been updated.

